# Ambient temperature and the occurrence of intradialytic hypotension in patients receiving hemodialysis

**DOI:** 10.1093/ckj/sfad304

**Published:** 2023-12-21

**Authors:** Kuan-Hung Liu, Wei-Hsiang Chang, Edward Chia-Cheng Lai, Pei-Chen Tsai, Bin Hsu, Yu-Hsuan Yang, Wei-Ren Lin, Tzu-Shan Huang, Fang-Yi Su, Jung-Hsien Chiang, Chung-Yi Li, Yau-Sheng Tsai, Junne-Ming Sung

**Affiliations:** Institute of Clinical Medicine, College of Medicine, National Cheng Kung University, Tainan, Taiwan; Division of Nephrology, Department of Internal Medicine, National Cheng Kung University Hospital, College of Medicine, National Cheng Kung University, Tainan, Taiwan; Department of Food Safety/ Hygiene and Risk Management, College of Medicine, National Cheng Kung University, Tainan, Taiwan; Research Center of Environmental Trace Toxic Substances, National Cheng Kung University, Tainan, Taiwan; School of Pharmacy, Institute of Clinical Pharmacy and Pharmaceutical Sciences, College of Medicine, National Cheng Kung University, Tainan, Taiwan; Department of Computer Science and Information Engineering, National Cheng Kung University, Tainan, Taiwan; Division of Nephrology, Department of Internal Medicine, National Cheng Kung University Hospital, College of Medicine, National Cheng Kung University, Tainan, Taiwan; Institute of Clinical Medicine, College of Medicine, National Cheng Kung University, Tainan, Taiwan; Institute of Clinical Medicine, College of Medicine, National Cheng Kung University, Tainan, Taiwan; Division of Nephrology, Department of Internal Medicine, National Cheng Kung University Hospital, College of Medicine, National Cheng Kung University, Tainan, Taiwan; Division of Nephrology, Department of Internal Medicine, National Cheng Kung University Hospital, College of Medicine, National Cheng Kung University, Tainan, Taiwan; Department of Computer Science and Information Engineering, National Cheng Kung University, Tainan, Taiwan; Department of Computer Science and Information Engineering, National Cheng Kung University, Tainan, Taiwan; Department of Public Health, College of Medicine, National Cheng Kung University, Tainan, Taiwan; Department of Public Health, College of Public Health, China Medical University, Taichung, Taiwan; Department of Healthcare Administration, College of Medical and Health Science, Asia University, Taichung, Taiwan; Institute of Clinical Medicine, College of Medicine, National Cheng Kung University, Tainan, Taiwan; Institute of Clinical Medicine, College of Medicine, National Cheng Kung University, Tainan, Taiwan; Division of Nephrology, Department of Internal Medicine, National Cheng Kung University Hospital, College of Medicine, National Cheng Kung University, Tainan, Taiwan

**Keywords:** ambient temperature, hemodialysis, intradialytic hypotension, seasonal variation, threshold

## Abstract

**Background:**

Intradialytic hypotension (IDH) is a common hemodialysis complication causing adverse outcomes. Despite the well-documented associations of ambient temperatures with fluid removal and pre-dialysis blood pressure (BP), the relationship between ambient temperature and IDH has not been adequately studied.

**Methods:**

We conducted a cohort study at a tertiary hospital in southern Taiwan between 1 January 2016 and 31 October 2021. The 24-h pre-hemodialysis mean ambient temperature was determined using hourly readings from the weather station closest to each patient's residence. IDH was defined using Fall40 [systolic BP (SBP) drop of ≥40 mmHg] or Nadir90/100 (SBP <100 if pre-dialysis SBP was ≥160, or SBP <90 mmHg). Multivariate logistic regression with generalizing estimating equations and mediation analysis were utilized.

**Results:**

The study examined 110 400 hemodialysis sessions from 182 patients, finding an IDH prevalence of 11.8% and 10.4% as per the Fall40 and Nadir90/100 criteria, respectively. It revealed a reverse J-shaped relationship between ambient temperature and IDH, with a turning point around 27°C. For temperatures under 27°C, a 4°C drop significantly increased the odds ratio of IDH to 1.292 [95% confidence interval (CI) 1.228 to 1.358] and 1.207 (95% CI 1.149 to 1.268) under the Fall40 and Nadir90/100 definitions, respectively. Lower ambient temperatures correlated with higher ultrafiltration, accounting for about 23% of the increased IDH risk. Stratified seasonal analysis indicated that this relationship was consistent in spring, autumn and winter.

**Conclusion:**

Lower ambient temperature is significantly associated with an increased risk of IDH below the threshold of 27°C, irrespective of the IDH definition. This study provides further insight into environmental risk factors for IDH in patients undergoing hemodialysis.

## Graphical Abstract

**Graphical Abstract:**
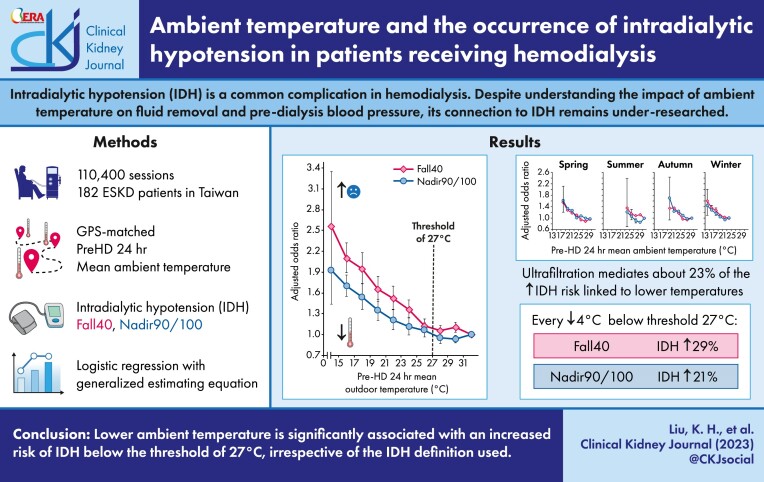

KEY LEARNING POINTS **What was known:**

- Intradialytic hypotension (IDH) is recognized as a common and potentially fatal complication during hemodialysis, yet there is no consensus on its definition.
- Ambient temperatures are linked to blood pressure and ultrafiltration, both closely related to IDH, though not conclusively studied in this context.

**This study adds:**

- The research demonstrates a reverse J-shaped relationship between ambient temperature and IDH, identifying approximately 27°C as a critical threshold, consistent across multiple IDH definitions.
- Below 27°C, each 4°C decrease in temperature is associated with up to a 29% increased risk of IDH.
- Lower temperatures before hemodialysis are associated with higher ultrafiltration, which mediates 23% of the cold temperature's impact on IDH.

**Potential impact:**

- Clinicians should consider environmental temperature as a factor in the care of hemodialysis patients and utilize optimal fluid management to partially mitigate cold-related IDH.
- Coping strategies and physiological mechanisms underlying low-temperature risks necessitate further research as they are critical to understanding and addressing IDH.

## INTRODUCTION

As climate change intensifies heat and cold, evaluating the potential health impacts of temperature exposure is crucial [1, 2]. Ambient temperature has been linked to an increased risk of cardiovascular morbidity and mortality [3–7]. Literature has reported that patients with end-stage kidney disease are susceptible to the hazards of temperature exposure [8, 9], partly relating to their high burden of cardiovascular disease and comorbidities [10].

Intradialytic hypotension (IDH), a common complication of hemodialysis, is associated with vascular access dysfunction [11], inadequate hemodialysis [12], morbidities [13–15] and mortality [13, 14, 16, 17]. Identification of associated risk factors is vital for improved patient care [14, 18–21]. However, the potential impact of ambient temperature on IDH remains understudied, despite these patients being particularly vulnerable to temperature challenges.

Cold temperatures affect blood pressure (BP) and water balance, with prior studies showing that dialysis patients in cold conditions have more fluid removal [ultrafiltration (UF)] and vasoconstriction [22, 23]. This leads to higher pre-dialysis BP and a diminished ability to counter fluid deficits, potentially causing IDH [22, 24].

Moreover, vasoconstriction may increase the heart's workload and oxygen demand, potentially disrupting steady circulation in susceptible patients [25]. Although low temperatures are linked to high UF and higher pre-dialysis BP—both risk factors for IDH—studies investigating the impact of ambient temperature on IDH are limited. Furthermore, previous studies on the health effects of temperature had methodological weaknesses; for example, they used ambient temperature data from a single weather station near the hemodialysis center or nearby airport, which may not accurately reflect patients’ actual residential temperature exposure [8, 9, 22, 26, 27].

In this study, we examined the association between 24-h mean ambient temperature obtained from Global Positioning System (GPS)-matched weather stations prior to each hemodialysis session. The objectives of the analysis were to (i) examine the correlation between ambient temperature and IDH; (ii) determine whether there is a threshold ambient temperature associated with IDH; (iii) identify the IDH definition most relevant to ambient temperature; and (iv) investigate any potential seasonal variations in this association. Findings from this study would contribute to understanding the potential influence of ambient temperature on IDH, which may aid in developing interventions to ameliorate the adverse effects of low temperatures on patients undergoing hemodialysis.

## MATERIALS AND METHODS

### Study design

**Figure 1:**
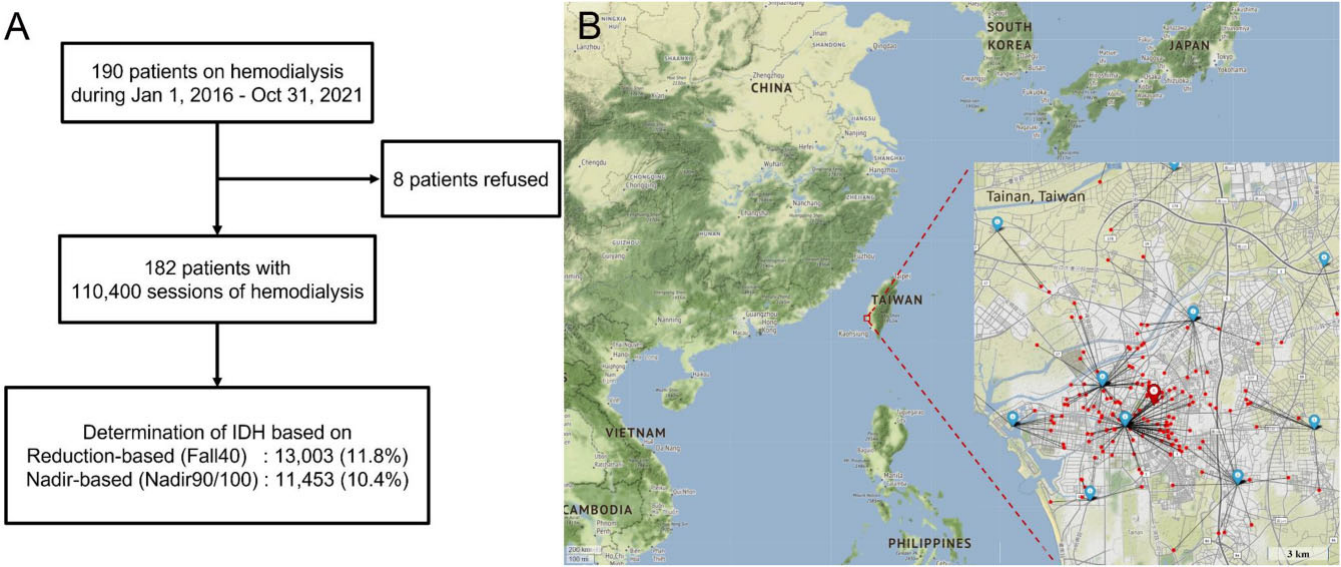
(**A**) Patient enrollment and geographic information. Patient enrollment and prevalence of IDH, (**B**) geographic information of southern Taiwan and the distance between the 21 weather stations of the CWB (blue marks) and the 182 home addresses assessed (red dots). Only 10 weather stations were shown in this figure due to the scale of figure. The dialysis center is shown as a red mark. The figure was generated by Python 3.8.9 with the extension library, Folium 0.12.1.

The Assessing Temperature Effect on Hemodialysis (ATEMPT-HD) study was an observational cohort study designed to investigate the association between temperature and hemodialysis outcomes. The study was conducted at the hemodialysis center of National Cheng Kung University Hospital, a tertiary teaching hospital in Tainan, Taiwan. In the study period between 1 January 2016 and 31 October 2021, a total of 190 patients underwent dialysis. Of these, 182 patients (95.8%) were included in the study analysis, including 17 deceased patients. Written informed consent was obtained from all living patients. The study was approved by the Institutional Review Board of National Cheng Kung University Hospital (A-ER-110-327) and adhered to the principles of the Declaration of Helsinki. The number of patients and the corresponding number of hemodialysis sessions are depicted in Fig. 1A.

### Temperature assessment

**Figure 2:**
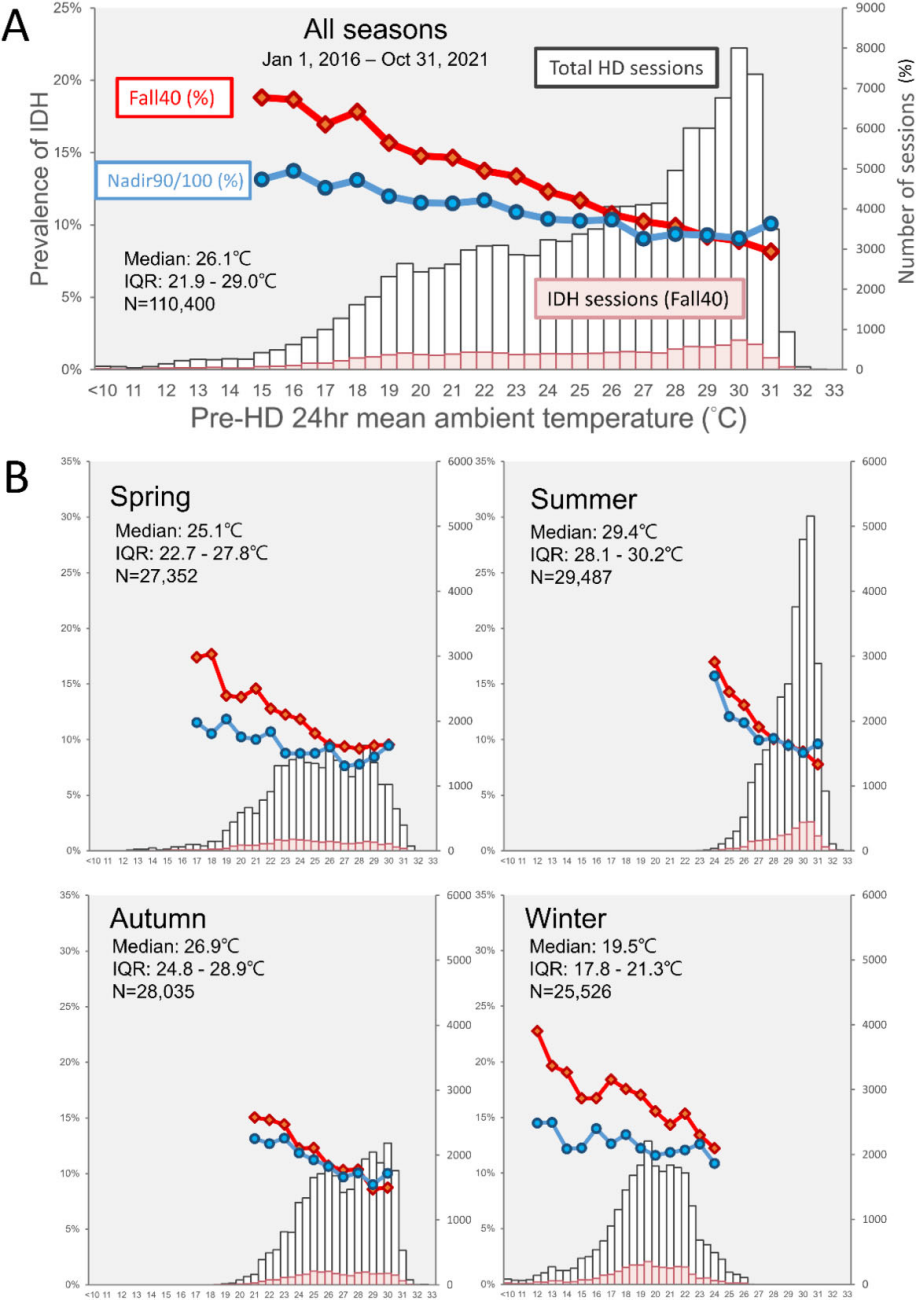
Distribution of mean ambient temperature during all hemodialysis sessions, both with and without IDH (in a stacked bar chart, refer to the right vertical axis for session numbers) and the prevalence of IDH in specific temperatures (in a line chart, refer to the left vertical axis) (**A**) for the entire observation period from 1 January 2016 to 31 October 2021, or (**B**) broken down by four seasons.

We obtained hourly ambient temperature measurements from the Central Weather Bureau (CWB) of Taiwan (https://opendata.cwb.gov.tw). The address of each patient's home was inserted into the GPS and coordinated according to its latitude and longitude. The closest weather station was determined based on the shortest great circle distance, calculated using the Haversine formula [28]. Temperature data were obtained from the weather station nearest to the patient's address (Fig. 1B). A total of 56 weather stations were affiliated with CWB in southern Taiwan (the study area), and 21 of them provided temperature readings for this study (Supplementary data, Table S1). We extracted the hourly temperature records for the 24 h preceding each hemodialysis session and calculated the mean ambient temperature over this period (i.e. pre-HD 24-h mean ambient temperature). The selection of 24 h is based on literature, which has shown that cold temperatures impact mortality with the strongest association on the same day [29]. Temperature distribution during hemodialysis sessions is shown in Fig. 2A and by season in Fig. 2B. IDH prevalence is also depicted based on specific temperatures.

### Determination of IDH

The determination of IDH was based on multiple criteria in the primary and sensitivity analyses. We adopted the reduction-based criteria (Fall40), which defines IDH as a reduction in systolic BP (SBP) of ≥40 mmHg (pre-HD SBP minus minimum SBP, ≥40 mmHg). We also used the nadir-based criteria (Nadir90/100), which defines IDH as the occurrence of intradialytic SBP <90 mmHg in patients with a pre-HD SBP <160 mmHg or SBP <100 mmHg in patients with a pre-HD SBP ≥160 mmHg. Moreover, we conducted sensitivity analyses with another five definitions of IDH to confirm the temperature–IDH relationship. Detailed definitions of IDH are shown in Supplementary data, Table S2. BP was measured before and every 60 min during hemodialysis, resulting in at least five records per 4-h session. Each session was then classified as either IDH or non-IDH. The prevalence of IDH according to the definitions of IDH is shown in Supplementary data, Table S2.

### Clinical covariates and dialysis records

Clinical covariates were collected and categorized into three groups: demographic and comorbidity information, hemodialysis records of each session and antihypertensive medications. Demographic and comorbidity data, such as age, sex, diabetes mellitus, hypertension, coronary artery disease and Charlson Comorbidity Index [30] were obtained by reviewing patient medical charts. Hemodialysis records of each session included information such as hemodialysis vintage (months), pre-HD SBP (mmHg), pre-HD body weight (kg), dry weight (kg), UF weight (kg), blood flow (mL/min), dialysate flow (mL/min), dialysate average temperature (°C), conductivity [dialysate sodium (mEq/L)], dialysate calcium (mEq/L) and monthly hemoglobin; all of these were extracted from the hospital electronic medical records. The UF weight to dry weight ratio (UF/DW) was calculated and presented as a percentage (%). Antihypertensive medications were categorized monthly into five groups: angiotensin-converting enzyme inhibitors/angiotensin receptor blockers, beta-blockers, alpha-blockers, calcium channel blockers and vasodilators. Additionally, data on the use of intravenous iron supplements (IV_iron) were extracted (see Supplementary data, Table S3). Under the Taiwan National Health Insurance program, prescriptions for more than one drug in the same category are restricted. Therefore, we coded each category as either present or absent.

### Statistical analyses

Data were presented as mean ± standard deviation (SD) or median with interquartile range for continuous variables and numbers and percentages for categorical variables. Characteristics of hemodialysis sessions were compared using standardized difference, a measure that is not sensitive to sample size [31]. A difference <0.1 was considered negligible.

The risk of IDH associated with ambient temperature was assessed using binary logistic regression. To account for the inter-correlations among hemodialysis sessions of the same patient, we applied the generalizing estimating equation (GEE) method with the first-order autoregressive (AR-1) as the working correlation matrix. The odds ratio (OR) for risk of IDH per four degrees (around 1 SD) in temperature was presented, considering the SD of the pre-HD 24-h mean ambient temperature was 4.4°C.

Sequential adjustments for covariates were made in the multivariable logistic regression models. We plotted the trend of crude and covariate-adjusted ORs of IDH per 2°C increase (0.5 SD of temperature) to visualize the temperature–IDH relationship and determine whether a threshold existed. If a threshold was identified, we further fitted and tested the interaction term of “above and below the threshold” and “pre-HD 24-hour mean ambient temperature.” A mediation analysis assessed the effects of temperature and UF/DW on IDH, adjusting for covariates. It used patient-specific random effects for repeated measures and Quasi-Bayesian confidence intervals (CIs) from 1000 bootstrap simulations [32].

To assess whether season may modify the effect of ambient temperature on IDH, we conducted a test for interaction between season and ambient temperature. Subsequently, subgroup analyses were conducted to assess the association of temperature with IDH by season. Seasons were categorized as spring (March–May), summer (June–August), autumn (September–November) and winter (December–February). Statistical analysis was conducted using SPSS and R. A *P*-value <.05 was considered statistically significant.

## RESULTS

### Prevalence of IDH in sessions by multiple definitions

We analyzed 110 400 hemodialysis sessions from 182 patients with 611 136 BP records. The prevalence of IDH in these sessions was estimated at 11.8% and 10.4% based on the reduction-based (Fall40) and nadir-based (Nadir90/100) criteria, respectively (Fig. 2A, Supplementary data, Table S2). The prevalence of IDH varied with ambient temperature, and the lowest and highest estimates were observed in hot (>31°C) and cold temperatures (<15°C) regardless of the IDH definition: 8.2% and 18% for Fall40 and 10.1% and 13.1% for Nadir90/100, respectively (Fig. 2A).

### Characteristics of IDH sessions

The differences in session characteristics and medication use between sessions with IDH and without IDH are shown in Table 1. The characteristic with the greatest standardized difference was pre-HD SBP for both definitions. Interestingly, pre-HD SBP was higher in IDH sessions defined by the Fall40 criteria (155 in IDH vs 134 mmHg in non-IDH) but was lower (121 in IDH vs 138 mmHg in non-IDH) in IDH sessions determined by Nadir90/100. IDH sessions also tended to have higher UF/DW than non-IDH sessions for both criteria, 4.91% vs 3.61% in Fall40 and 4.63% vs 3.66% in Nadir90/100. Age and sex were also differently distributed between the definitions, with IDH sessions defined by Fall40 being younger and male, and IDH sessions defined by Nadir90/100 being older and female (Table 1).

**Table 1:** Characteristics of hemodialysis sessions according to IDH definitions.

			Reduction-based (Fall40)			Nadir-based (Nadir90/100)		
Variable	Total number	All sessions	Non-IDH (*n* = 97 397)	IDH (*n* = 13 003)	Standardized difference	Non-IDH (*n* = 98 947)	IDH (*n* = 11 453)	Standardized difference
Pre-HD 24 h. mean temp. (°C), mean (SD)	110 400	25.2 (4.4)	25.3 (4.3)	24.1 (4.7)	0.272	25.2 (4.4)	24.7 (4.6)	0.128
Demographic data								
Age (years), mean (SD)	110 400	62.9 (13.7)	62.9 (13.7)	63.3 (13.3)	0.035	62.3 (13.6)	68.6 (13.4)	0.470
Male, *n* (%)	110 400	55 644 (50.4)	47 837 (49.1)	7807 (60.0)	0.221	51 518 (52.1)	4126 (36.0)	0.327
DM, *n* (%)	110 400	48 180 (43.6)	40 020 (41.1)	8160 (62.8)	0.444	42 179 (42.6)	6001 (52.4)	0.197
HTN, *n* (%)	110 400	83 010 (75.2)	72 725 (74.7)	10 285 (79.1)	0.105	75 602 (76.4)	7408 (64.7)	0.259
CAD, *n* (%)	110 400	27 222 (24.7)	24 380 (25.0)	2842 (21.9)	0.075	24 163 (24.4)	3059 (26.7)	0.052
CCI, mean (SD)	110 400	7.0 (2.8)	7.0 (2.9)	7.4 (2.6)	0.146	6.9 (2.8)	8.0 (2.7)	0.407
Hemodialysis records								
HD vintage (months), mean (SD)	110 400	59 (53)	60 (54)	52 (46)	0.163	59 (53)	62 (58)	0.050
UF/DW (%), mean (SD)	108 635	3.76 (1.80)	3.61 (1.74)	4.91 (1.85)	0.726	3.66 (1.77)	4.63 (1.83)	0.539
Pre-HD SBP (mmHg), mean (SD)	110 395	136 (22)	134 (21)	155 (22)	0.987	138 (21)	121 (27)	0.702
Blood flow (mL/min), mean (SD)	110 084	275 (36)	275 (35)	277 (38)	0.040	277 (35)	260 (34)	0.483
Dialysate flow (mL/min), mean (SD)	110 379	564 (113)	560 (110)	597 (123)	0.317	566 (114)	547 (100)	0.176
Dialysate avg. temp. (°C), mean (SD)	110 231	36.1 (0.4)	36.1 (0.4)	36.0 (0.4)	0.244	36.1 (0.4)	35.9 (0.4)	0.448
Conductivity (mEq/L), mean (SD)	109 769	140.0 (0.6)	140.0 (0.6)	140.0 (0.7)	0.031	140.0 (0.7)	140.0 (0.5)	0.033
Dialysate calcium (mEq/L), *n* (%)	110 384							
1.8		1490 (1.3)	1336 (1.4)	154 (1.2)	0.017	1428 (1.4)	62 (0.5)	0.091
2.5		58 946 (53.4)	52 021 (53.4)	6925 (53.3)	0.003	52 712 (53.3)	6234 (54.4)	0.023
3.0		42 286 (38.3)	37 230 (38.2)	5056 (38.9)	0.014	37 755 (38.2)	4531 (39.6)	0.029
3.5		7662 (6.9)	6794 (7.0)	868 (6.7)	0.012	7037 (7.1)	625 (5.5)	0.068
Hb, mean (SD)	110 374	10.6 (1.0)	10.6 (1.0)	10.8 (1.0)	0.188	10.6 (1.0)	10.7 (1.0)	0.150
Antihypertensives and iron use, *n* (%)	107 867							
ACEi/ARB		27 204 (25.2)	24 320 (25.0)	2884 (22.2)	0.066	26 216 (26.5)	988 (8.6)	0.483
Beta-blockers		31 692 (29.4)	27 943 (28.7)	3749 (28.8)	0.003	29 755 (30.1)	1937 (16.9)	0.314
Alpha-blockers		11 912 (11.0)	10 823 (11.1)	1089 (8.4)	0.092	11 530 (11.7)	382 (3.3)	0.320
Calcium channel blocker		40 708 (37.7)	37 099 (38.1)	3609 (27.8)	0.221	39 594 (40.0)	1114 (9.7)	0.748
Vasodilator		8712 (8.1)	7951 (8.2)	761 (5.9)	0.091	8289 (8.4)	423 (3.7)	0.198
IV_iron	110 400	1569 (1.4)	1392 (1.4)	177 (1.4)	0.000	1457 (1.5)	112 (1.0)	0.158

Data presented as mean (SD) in the continuous variables, and numbers (*n*) with the percentage (%) in categorical variables.

A standardized difference of <0.1 was considered a negligible difference between groups.

Temp., temperature; DM, diabetes mellitus; HTN, hypertension; CAD, coronary artery disease; CCI, Charlson Comorbidity Index; HD, hemodialysis; avg, average; Hb, hemoglobin; ACEi, angiotensin-converting enzyme inhibitor; ARB, angiotensin-receptor blocker; IV_iron, intravenous iron infusion.

### Relationship between ambient temperature and IDH

**Figure 3:**
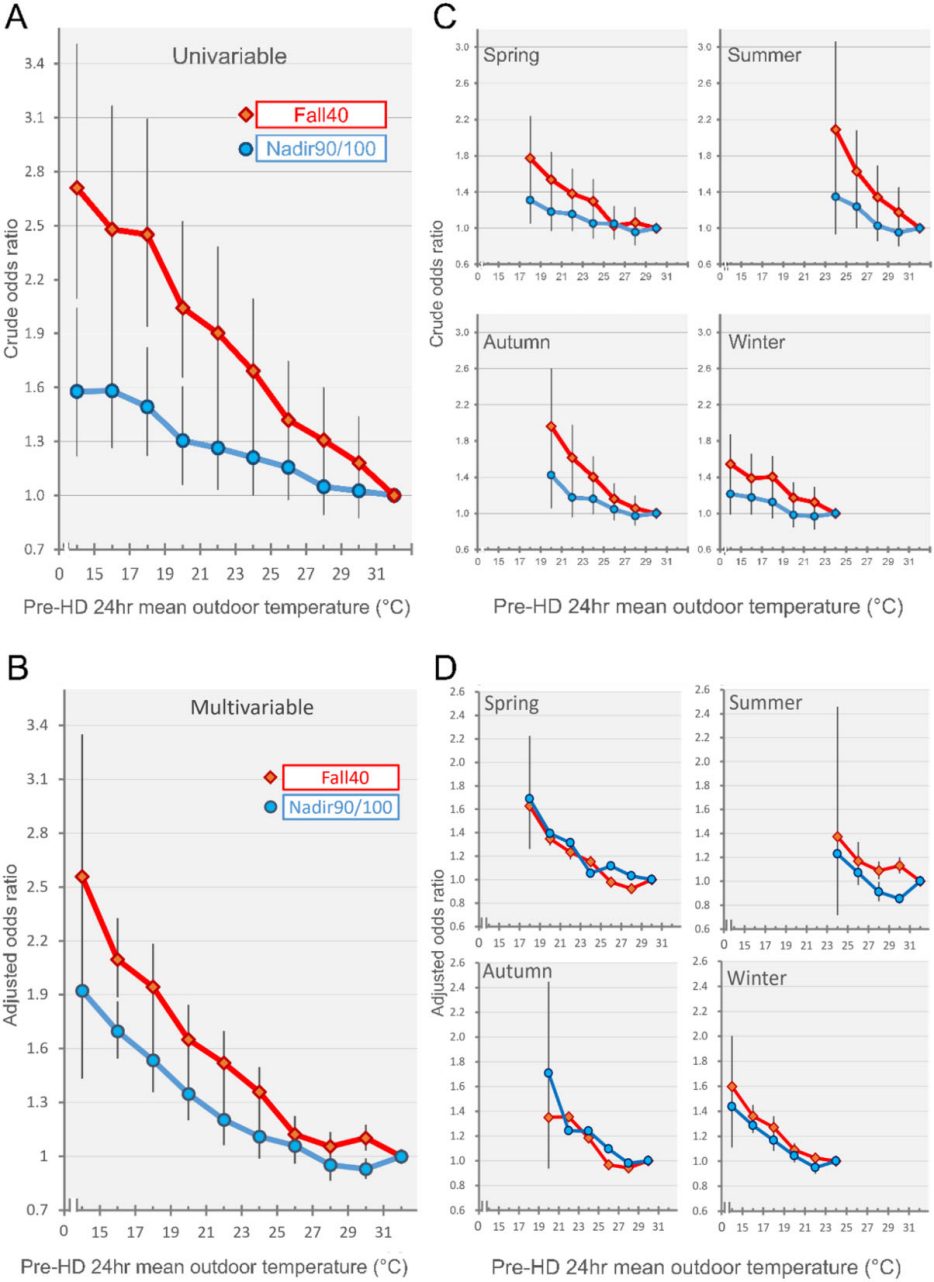
Relationship between ambient temperature and risk of IDH compared with the subsets of highest temperature. (**A**) displays the crude OR, while (**B**) shows the adjusted OR with adjustments made for age, sex, diabetes, coronary artery disease, Charlson Comorbidity Index, UF/DW, pre-HD SBP, blood flow, dialysate flow, dialysate mean temperature, conductivity, dialysate calcium, hemoglobin and antihypertensive medication use. Additionally, the relationship between environmental temperature and the risk of IDH is further examined by season in (**C**) and (**D**), with crude and adjusted ORs, respectively, using the same adjustments as in (B).

We observed that the relationship between ambient temperature and IDH was not linear but exhibited a reverse J-shaped pattern, indicating a possible inflection point around 27°C. The linear relationship was found only in temperatures below 27°C (Fig. 3A and B). We performed a test for interaction and found significant results with *P-*values of .001 and .033 for both Fall40 and Nadir90/100 definitions, suggesting that the effect of temperature on the risk of IDH was significantly different between temperatures above and below 27°C (Table 2). However, using 26°C and 28°C as alternative cut-off points, the Nadir90/100 definition showed no consistent, significant interaction. We then conducted stratified analyses. For temperatures above 27°C, the ORs of IDH were 0.924 (95% CI 0.772 to 1.105) for the Fall40 definition and 1.010 (95% CI 0.830 to 1.228) for the Nadir90/100 definition. In contrast, for temperatures below 27°C, the OR of IDH per 4°C decrease was significantly increased at 1.292 (95% CI 1.228 to 1.358) and 1.207 (95% CI 1.149 to 1.268) for the Fall40 and Nadir90/100 definitions, respectively. Therefore, all subsequent analyses were limited to temperatures <27°C.

**Table 2:** Logistic regression with GEE methods for risk of IDH per 4°C decrease above and below the threshold (27°C).

			Stratified by temperature		
		Overall	≤27°C	>27°C	
Definition		(*n* = 105 895)^a^	(*n* = 59 751)^a^	(*n* = 46 144)^a^	*P*_interaction_^b^
Fall40	Adjusted^c^ OR	1.230	1.292	0.924	
	95% CI	1.175–1.289	1.228–1.358	0.772–1.105	
	*P*-value	<.001	<.001	.39	.001
Nadir90/100	Adjusted^c^ OR	1.181	1.207	1.010	
	95% CI	1.126–1.238	1.149–1.268	0.830–1.228	
	*P*-value	<.001	<.001	.92	.033

a The number was reduced because of missing data.

b Interaction for ambient temperature and temperature above/below the threshold 27°C.

c Adjusted for age, sex, diabetes, coronary artery disease, CCI, HD vintage, UF/DW, pre-HD SBP, blood flow, dialysate flow, dialysate average temperature, conductivity, dialysate calcium, hemoglobin, antihypertensive medications and iron infusion.

### Temperature–IDH relationship after multivariable adjustment

In temperatures below the threshold of 27°C, we conducted univariate and multivariable logistic regression analyses with adjustments for demographic data, dialysis-related variables and medications in Models 1–3 accordingly (Table 3). With IDH defined by Fall40, the crude and adjusted ORs were similar across the univariate and adjusted Models 1–3 with a change in OR <3%. Conversely, the OR increased from 1.123 in the crude OR to 1.207 in the fully adjusted OR, representing a 7% increase if IDH was defined by Nadir90/100. The fully adjusted models showed that each 4°C decrease from 27°C to 6.2°C was associated with an adjusted OR of 1.292 (95% CI 1.228 to 1.358) for Fall40 and 1.207 (95% CI 1.149 to 1.268) for Nadir90/100.

**Table 3:** Univariate and multivariable logistic regression with GEE methods for risk of IDH per 4°C decrease below the threshold (27°C).

Definition		Univariate	Model 1^a^	Model 2^b^	Model 3^c^
Fall40	OR	1.244	1.257	1.273	1.292
	95% CI	1.186–1.305	1.197–1.319	1.210–1.338	1.228–1.358
	*P*-value	<.001	<.001	<.001	<.001
Nadir90/100	OR	1.123	1.121	1.185	1.207
	95% CI	1.078–1.169	1.074–1.170	1.131–1.242	1.149–1.268
	*P*-value	<.001	<.001	<.001	<.001

a Model 1 adjusted for age, sex, diabetes, hypertension, coronary artery disease and Charlson Comorbidity Index.

b Model 2 adjusted for covariates in Model 1 and additionally adjusted for hemodialysis vintage, UF/DW, pre-HD SBP, blood flow, dialysate flow, dialysate average temperature, conductivity and dialysate calcium.

c Model 3 further adjusted for antihypertensive medications and iron infusion, but omitted covariate of hypertension to avoid collinearity.

### Causal mediation analysis

Fluid removal (UF/DW) substantially mediated the effect of temperature on IDH risk, accounting for 23.16% (95% CI 21.23% to 25.40%) of the total effect (Supplementary data, Table S4). Both direct and mediated effects were significant.

### Temperature effect on IDH across the four seasons

Analysis of interaction indicates that season significantly modifies the relationship between ambient temperature and IDH, according to the covariate-adjusted Nadir90/100 criteria (interaction *P*-value = .029). However, this interaction is not significant when assessed using the Fall40 criteria (interaction *P*-value = .728). After stratifying by four seasons, the low ambient temperature remained significantly associated with IDH in all seasons except summer. The adjusted ORs (95% CIs) of IDH defined by Fall40 were 1.267 (1.167 to 1.375), 0.863 (0.440 to 1.694), 1.368 (1.176 to 1.591) and 1.227 (1.136 to 1.325) in the spring, summer, autumn and winter, respectively. The corresponding ORs for the Nadir90/100 definition were 1.251 (1.134 to 1.381), 0.886 (0.409 to 1.919), 1.207 (1.010 to 1.443) and 1.182 (1.093 to 1.277) (Table 4, Fig. 3C and D).

**Table 4:** Univariate and multivariable logistic regression for risk of IDH per 4°C decrease in four seasons (≤27°C).

		Sessions with temperature ≤27°C (*n* = 62 234)							
		Unadjusted				Adjusted^a^			
Definition		Spring (*n* = 18 781)	Summer (*n* = 3565)	Autumn (*n* = 14 362)	Winter (*n* = 25 526)	Spring (*n* = 17 372)^b^	Summer (*n* = 3271)^b^	Autumn (*n* = 13 383)^b^	Winter (*n* = 23 259)^b^
Fall40	OR	1.278	1.260	1.415	1.189	1.267	0.863	1.368	1.227
	95% CI	1.196–1.366	0.753–2.108	1.232–1.625	1.119–1.264	1.167–1.375	0.440–1.694	1.176–1.591	1.136–1.325
	*P*-value	<.001	.38	<.001	<.001	<.001	.67	<.001	<.001
	*P*_interaction_^c^	.343				.728			
Nadir90/100	OR	1.136	0.884	1.158	1.110	1.251	0.886	1.207	1.182
	95% CI	1.050–1.229	0.510–1.533	1.006–1.333	1.047–1.177	1.134–1.381	0.409–1.919	1.010–1.443	1.093–1.277
	*P*-value	.002	.66	.041	<.001	<.001	.76	.038	<.001
	*P*_interaction_^c^	.134				.029			

a Adjusted for age, sex, diabetes, coronary artery disease, Charlson Comorbidity Index, hemodialysis vintage, UF/DW, pre-HD SBP, blood flow, dialysate flow, dialysate average temperature, conductivity, dialysate calcium, hemoglobin, antihypertensive medications and iron infusion.

b The number is reduced because of missing data.

c Interaction for season and ambient temperature.

### Sensitivity analysis

We conducted sensitivity analyses incorporating an additional five definitions outlined in Supplementary data, Table S2. The temperature–IDH relationship based on these definitions revealed similar patterns (Supplementary data, Fig. S1). The characteristics of hemodialysis sessions according to various IDH definitions in sensitivity analysis are shown in Supplementary data, Table S5. The threshold was evident in all definitions with *P*_interaction_ < .05 (Supplementary data, Table S6). We repeated multivariable logistic regression and found the ORs to be similar in magnitude to those obtained using the Fall40 and Nadir90/100 definitions outlined in Supplementary data, Table S7.

## DISCUSSION

The current study found that low ambient temperature is associated with an increased risk of IDH. More specifically, for every 4°C drop in temperature, the OR of IDH increased by 1.29 and 1.21 for the reduction-based and nadir-based definitions, respectively. Furthermore, our analysis revealed a statistically significant threshold of 27°C across all seven predetermined IDH definitions, with the reduction-based definition (Fall40) being particularly relevant for cold temperatures. Moreover, ambient temperature's impact on IDH was 23% mediated by changes in UF (UF/DW). The relationship between temperature and IDH was consistent across all seasons except summer. These results have important implications for the management and care of hemodialysis patients, as well as for developing interventions and policies related to cold weather exposure.

In this study, we identified a threshold temperature of 27°C for the temperature–IDH relationship in southern Taiwan. An analysis of mortality in 11 Eastern US cities revealed that temperature was the most significant weather-related factor affecting mortality. Southern cities, which have similar latitudes to Taiwan, had a higher risk of mortality at lower temperatures, while lower risk at higher temperatures [33]. The study found threshold temperatures of 27.06°C and 27.18°C in Tampa and Miami, similar to a previously identified threshold of 27°C for cardiovascular mortality in Taiwan [6]. Although the threshold temperature associated with IDH risk may vary in different regions, the findings from our study might be generalized to regions with similar latitudes. Further studies across different regions and latitudes are needed.

The IDH definition is currently lacking consensus [13, 14, 34]. We used two primary definitions, both of which were reported to have a significant association with mortality in different patient populations. Shoji *et al*. [17] found that a reduction in systolic blood pressure SBP of more than 40 mmHg (Fall40) was associated with 2-year mortality in a Japanese population. Flythe *et al*. [16] reported that Nadir90/100 was consistently associated with mortality in two large cohorts in the USA. Our analysis found Fall40 to be more strongly associated with cold temperatures. Cold temperatures are associated with high UF rates, which are a risk factor for SBP decline and thus more likely to meet the criteria of Fall40. In addition, cold temperatures are linked to higher pre-HD SBP [35, 36], which may make individuals less likely to reach Nadir90/100 criteria, as the lowest intradialytic SBP determines it. Our results suggest that Fall40 is more sensitive to cold ambient temperatures. The potential impact of different definitions on the relationship between IDH and temperature should be further explored in future studies.

Previous research has shown that there are seasonal variations in hemodialysis initiation [37], reductions in intradialytic BP [27], heart failure [38, 39], acute myocardial infarctions [40] and overall mortality [41], with the highest risks observed in winter. Our study differentiates between “winter” and “low temperatures” as they are related but not contextually identical. We conducted a stratified analysis to examine the difference in the temperature–IDH relationship in four seasons. The results indicate that cold temperature remained a risk factor for IDH across seasons in spring, autumn and winter. This provided reassurance that certain season-related factors such as lifestyle, comorbidity and diet would not confound the relationship obverted in this study. In addition, such findings also challenged the perception that winter is the only season associated with an increased risk of IDH (Table 4, Fig. 3D).

The analysis showed no significant association in the summer, possibly due to the hot and stable weather of southern Taiwan's summer, which has a small temperature variation with an interquartile range of around 2.1°C (Fig. 2B). This lack of temperature variation may facilitate temperature acclimation, potentially reducing the risk of IDH [42–45]. Interestingly, the spring and autumn seasons had the highest adjusted ORs and most dispersed temperature distribution, aligning with the idea of temperature acclimation.

Our findings demonstrate a correlation between lower temperatures and increased IDH risk, with a negative relationship between temperature and UF (UF/DW) (Supplementary data, Fig. S2). Mediation analysis found that approximately one-quarter of the IDH risk from cold temperature was attributable to increased UF, suggesting that controlling UF volumes may offer an opportunity to partially mitigate the influence of low temperatures on IDH (Supplementary data, Table S4). Besides, previous research has established the efficacy of indoor heating as a means of combating cold outdoor temperatures. One randomized controlled trial evaluated the effect of intensive room heating on BP in healthy Japanese participants during the winter months [46]. The results showed that intensive heating was associated with a significant reduction in morning SBP and a suppression of morning pressure surges compared with weak heating. However, further research is needed to ascertain the potential benefits of this intervention for dialysis patients.

Our study has several strengths. First, the large sample size of over 110 000 dialysis sessions improves the assessment of temperature exposure. The study matched each patient to temperature data from the nearest weather station. Second, we have collected detailed parameters of dialysis, such as UF, dialysate temperature, dialysate calcium and hemodialysis vintage, which are all important factors related to IDH. Adjustments for these parameters largely reduced the potential for confounding. Third, as most dialysis patients have hypertension, we adjusted for antihypertensive medications, which may significantly impact IDH. Fourth, we included living and deceased patients from our dialysis center, reducing the potential selection and survivorship bias. Lastly, the temporal relationship between temperature exposure and IDH is clear, as the temperature is traced back according to the timing of each session.

Despite all the efforts, this study has limitations. One limitation is that we could only investigate ambient temperature, not indoor temperature. However, a prior study found a strong correlation between outdoor and indoor temperatures when the outdoor temperature exceeded 12.7°C [47]. Moreover, when cold waves strike, the actual temperature patients are exposed to indoors may not be as cold as the recorded outdoor temperature, introducing potential exposure misclassification. This could potentially bias our results toward the null, however the study still presents significant findings. While this study incorporates UF normalized to dry weight (UF/DW), the inclusion of bioimpedance measurements would offer a more detailed representation of body fluid changes, which can be considered in future studies. Additionally, there may be confounding factors, such as patients’ adaptive behaviors to cold, that were not accounted for. However, we believe that stratifying the data by season has partially controlled for these variables. Another limitation is that this study was conducted in Tainan, Taiwan, a subtropical climate city. Therefore, it is unclear whether the findings apply to places with different climates or latitudes. Further research is needed to assess the generalizability of the findings.

## CONCLUSION

Our study provides important insights into the relationship between low ambient temperature and IDH. Low ambient temperature before hemodialysis is associated with an increased risk of IDH regardless of IDH definitions. Future studies should investigate the potential benefits of interventions such as indoor heating for dialysis patients and the generalizability of these findings to other latitudes and climates. In addition, clinicians should consider environmental temperature a potential risk factor in patient care.

## Supplementary Material

sfad304_Supplemental_File

## Data Availability

The data sharing is beyond the scope of the authorization from the patient's informed consent and from the Institutional Review Board.

## References

[1] Deschenes O. Temperature, human health, and adaptation: a review of the empirical literature. Energy Econ 2014;46:606–19. 10.1016/j.eneco.2013.10.013

[2] McMichael AJ. Globalization, climate change, and human health. N Engl J Med 2013;368:1335–43. 10.1056/NEJMra1109341 23550671

[3] Bai L , Li Q, Wang J et al. Increased coronary heart disease and stroke hospitalisations from ambient temperatures in Ontario. Heart 2018;104:673–9. 10.1136/heartjnl-2017-311821 29101264 PMC5890650

[4] Cheng J , Su H, Xu Z et al. Extreme temperature exposure and acute myocardial infarction: elevated risk within hours? Environ Res 2021;202:111691. 10.1016/j.envres.2021.111691 34331920

[5] Fukuda H , Ninomiya H, Ueba Y et al. Impact of temperature decline from the previous day as a trigger of spontaneous subarachnoid hemorrhage: case-crossover study of prefectural stroke database. J Neurosurg 2019;133:374–82. 10.3171/2019.4.JNS19175 31277067

[6] Yang LT , Chang YM, Hsieh TH et al. Associations of ambient temperature with mortality rates of cardiovascular and respiratory diseases in Taiwan: a subtropical country. Acta Cardiol Sin 2018;34:166–74. 29643703 10.6515/ACS.201803_34(2).20171101A PMC5863071

[7] Mohammadi D , Zare Zadeh M, Zare Sakhvidi MJ. Short-term exposure to extreme temperature and risk of hospital admission due to cardiovascular diseases. Int J Environ Health Res 2021;31:344–54. 10.1080/09603123.2019.1663496 33615930

[8] Remigio RV , Turpin R, Raimann JG et al. Assessing proximate intermediates between ambient temperature, hospital admissions, and mortality in hemodialysis patients. Environ Res 2022;204:112127. 10.1016/j.envres.2021.112127 34582801 PMC8901270

[9] Remigio RV , Jiang C, Raimann J et al. Association of extreme heat events with hospital admission or mortality among patients with end-stage renal disease. JAMA Netw Open 2019;2:e198904. 10.1001/jamanetworkopen.2019.8904 31397862 PMC6692691

[10] Cozzolino M , Mangano M, Stucchi A et al. Cardiovascular disease in dialysis patients. Nephrol Dial Transplant 2018;33:iii28–34. 10.1093/ndt/gfy174 30281132 PMC6168816

[11] Chang TI , Paik J, Greene T et al. Intradialytic hypotension and vascular access thrombosis. J Am Soc Nephrol 2011;22:1526–33. 10.1681/ASN.2010101119 21803971 PMC3148707

[12] Ronco C , Brendolan A, Milan M et al. Impact of biofeedback-induced cardiovascular stability on hemodialysis tolerance and efficiency. Kidney Int 2000;58:800–8. 10.1046/j.1523-1755.2000.00229.x 10916105

[13] Sars B , van der Sande FM, Kooman JP. Intradialytic hypotension: mechanisms and outcome. Blood Purif 2020;49:158–67. 10.1159/000503776 31851975 PMC7114908

[14] Kanbay M , Ertuglu LA, Afsar B et al. An update review of intradialytic hypotension: concept, risk factors, clinical implications and management. Clin Kidney J 2020;13:981–93. 10.1093/ckj/sfaa078 33391741 PMC7769545

[15] Zoccali C , Benedetto FA, Tripepi G et al. Cardiac consequences of hypertension in hemodialysis patients. Semin Dial 2004;17:299–303. 10.1111/j.0894-0959.2004.17331.x 15250922

[16] Flythe JE , Xue H, Lynch KE et al. Association of mortality risk with various definitions of intradialytic hypotension. J Am Soc Nephrol 2015;26:724–34. 10.1681/ASN.2014020222 25270068 PMC4341481

[17] Shoji T , Tsubakihara Y, Fujii M et al. Hemodialysis-associated hypotension as an independent risk factor for two-year mortality in hemodialysis patients. Kidney Int 2004;66:1212–20. 10.1111/j.1523-1755.2004.00812.x 15327420

[18] Gul A , Miskulin D, Harford A et al. Intradialytic hypotension. Curr Opin Nephrol Hypertens 2016;25:545–50. 10.1097/MNH.0000000000000271 27606498

[19] Stefánsson BV , Brunelli SM, Cabrera C et al. Intradialytic hypotension and risk of cardiovascular disease. Clin J Am Soc Nephrol 2014;9:2124–32. 10.2215/CJN.02680314 25376764 PMC4255399

[20] Sands JJ , Usvyat LA, Sullivan T et al. Intradialytic hypotension: frequency, sources of variation and correlation with clinical outcome. Hemodial Int 2014;18:415–22. 10.1111/hdi.12138 24467830

[21] Davenport A. Why is intradialytic hypotension the commonest complication of outpatient dialysis treatments? Kidney Int Rep 2023;8:405–18. 10.1016/j.ekir.2022.10.031 36938081 PMC10014354

[22] Cheung AK , Yan G, Greene T et al. Seasonal variations in clinical and laboratory variables among chronic hemodialysis patients. J Am Soc Nephrol 2002;13:2345–52. 10.1097/01.ASN.0000026611.07106.A7 12191979

[23] Flythe JE , Assimon MM, Wenger JB et al. Ultrafiltration rates and the quality incentive program: proposed measure definitions and their potential dialysis facility implications. Clin J Am Soc Nephrol 2016;11:1422–33. 10.2215/CJN.13441215 27335126 PMC4974895

[24] Alba BK , Castellani JW, Charkoudian N. Cold-induced cutaneous vasoconstriction in humans: function, dysfunction and the distinctly counterproductive. Exp Physiol 2019;104:1202–14. 10.1113/EP087718 31045297

[25] Wilson TE , Gao Z, Hess KL et al. Effect of aging on cardiac function during cold stress in humans. Am J Physiol Regul Integr Comp Physiol 2010;298:R1627–33. 10.1152/ajpregu.00099.2010 20375268 PMC2886703

[26] Duranton F , Kramer A, Szwarc I et al. Geographical variations in blood pressure level and seasonality in hemodialysis patients. Hypertension 2018;71:289–96. 10.1161/HYPERTENSIONAHA.117.10274 29255071

[27] Uchiyama K , Shibagaki K, Yanai A et al. Seasonal variation and predictors of intradialytic blood pressure decline: a retrospective cohort study. Hypertens Res 2021;44:1417–27. 10.1038/s41440-021-00714-1 34331031

[28] Robusto CC. The Cosine-Haversine formula. Am Math Mon 1957;64:38–40. 10.2307/2309088

[29] Braga AL , Zanobetti A, Schwartz J. The time course of weather-related deaths. Epidemiology 2001;12:662–7. 11679794 10.1097/00001648-200111000-00014

[30] Charlson ME , Pompei P, Ales KL et al. A new method of classifying prognostic comorbidity in longitudinal studies: development and validation. J Chronic Dis 1987;40:373–83. 10.1016/0021-9681(87)90171-8 3558716

[31] Austin PC. Balance diagnostics for comparing the distribution of baseline covariates between treatment groups in propensity-score matched samples. Stat Med 2009;28:3083–107. 10.1002/sim.3697 19757444 PMC3472075

[32] Tingley D , Yamamoto T, Hirose K et al. mediation: R package for causal mediation analysis. J Stat Soft 2014;59:1–38. 10.18637/jss.v059.i05

[33] Curriero FC , Heiner KS, Samet JM et al. Temperature and mortality in 11 cities of the eastern United States. Am J Epidemiol 2002;155:80–7. 10.1093/aje/155.1.80 11772788

[34] Assimon MM , Flythe JE. Definitions of intradialytic hypotension. Semin Dial 2017;30:464–72. 10.1111/sdi.12626 28691195 PMC5668149

[35] Hartwig SV , de Souza Hacon S, de Oliveira BFA et al. The effect of ambient temperature on blood pressure of patients undergoing hemodialysis in the Pantanal-Brazil. Heliyon 2021;7:e07348. 10.1016/j.heliyon.2021.e07348 34235283 PMC8246300

[36] Wu Z , Lan S, Chen C et al. Seasonal variation: a non-negligible factor associated with blood pressure in patients undergoing hemodialysis. Front Cardiovasc Med 2022;9:820483. 10.3389/fcvm.2022.820483 35369290 PMC8971928

[37] Obi Y , Kalantar-Zadeh K, Streja E et al. Seasonal variations in transition, mortality and kidney transplantation among patients with end-stage renal disease in the USA. Nephrol Dial Transplant 2017;32:ii99–105. 10.1093/ndt/gfw379 28201764 PMC6557195

[38] Boulay F , Berthier F, Sisteron O et al. Seasonal variation in chronic heart failure hospitalizations and mortality in France. Circulation 1999;100:280–6. 10.1161/01.CIR.100.3.280 10411853

[39] Stewart S , McIntyre K, Capewell S et al. Heart failure in a cold climate. Seasonal variation in heart failure-related morbidity and mortality. J Am Coll Cardiol 2002;39:760–6. 10.1016/S0735-1097(02)01685-6 11869838

[40] Nagarajan V , Fonarow GC, Ju C et al. Seasonal and circadian variations of acute myocardial infarction: findings from the Get With The Guidelines-Coronary Artery Disease (GWTG-CAD) program. Am Heart J 2017;189:85–93. 10.1016/j.ahj.2017.04.002 28625385

[41] Usvyat LA , Carter M, Thijssen S et al. Seasonal variations in mortality, clinical, and laboratory parameters in hemodialysis patients: a 5-year cohort study. Clin J Am Soc Nephrol 2012;7:108–15. 10.2215/CJN.03880411 22096041 PMC3265352

[42] Daanen HA , Van Marken Lichtenbelt WD. Human whole body cold adaptation. Temperature 2016;3:104–18. 10.1080/23328940.2015.1135688 PMC4861193 27227100

[43] Yurkevicius BR , Alba BK, Seeley AD et al. Human cold habituation: physiology, timeline, and modifiers. Temperature 2022;9:122–57. 10.1080/23328940.2021.1903145 PMC9467574 36106151

[44] Leppäluoto J , Korhonen I, Hassi J. Habituation of thermal sensations, skin temperatures, and norepinephrine in men exposed to cold air. J Appl Physiol 2001;90:1211–8. 10.1152/jappl.2001.90.4.1211 11247916

[45] Stewart S , Keates AK, Redfern A et al. Seasonal variations in cardiovascular disease. Nat Rev Cardiol 2017;14:654–64. 10.1038/nrcardio.2017.76 28518176

[46] Saeki K , Obayashi K, Iwamoto J et al. Influence of room heating on ambulatory blood pressure in winter: a randomised controlled study. J Epidemiol Community Health 2013;67:484–90. 10.1136/jech-2012-201883 23447647

[47] Nguyen JL , Schwartz J, Dockery DW. The relationship between indoor and outdoor temperature, apparent temperature, relative humidity, and absolute humidity. Indoor Air 2014;24:103–12. 10.1111/ina.12052 23710826 PMC3791146

