# Supplementary material for: Ambient temperature and the occurrence of intradialytic hypotension in patients receiving hemodialysis
Source: Clin Kidney J. 2023 Dec 21;17(1):sfad304. doi: 10.1093/ckj/sfad304 (PMC10783262; doi:10.1093/ckj/sfad304)
Supplement: sfad304_Supplemental_File [file sfad304_supplemental_file.docx]

**Ambient Temperature and the Occurrence of Intradialytic Hypotension in Patients Receiving Hemodialysis**

Kuan-Hung Liu, MD^1,2^ Wei-Hsiang Chang, PhD^3,4^ Edward Chia-Cheng Lai, PhD^5^ Pei-Chen Tsai, MS^6^ Bin Hsu, MS^2^ Yu-Hsuan Yang, MS^1^ Wei-Ren Lin, MD^1,2^, Tzu-Shan Huang, MD^2^ Fang-Yi Su, MD^6^ Jung-Hsien Chiang, PhD^6^ Chung-Yi Li, PhD^7^ Yau-Sheng Tsai, PhD^1*^ and Junne-Ming Sung, MD, PhD^1,2*^

**Supplement.**

**Table S1.** The location of the extracted weather stations and session numbers of matched stations.

**Table S2.** Prevalence of IDH according to various definitions in primary and sensitivity analyses.

**Table S3.** List of anti-hypertensive medications and iron supplement analyzed in the study.

**Table S4.** Causal mediation of ambient temperature effect on intradialytic hypotension via ultrafiltration per dry weight.

**Table S5.:** Characteristics of HD sessions according to IDH definitions.

**Table S6.** Logistic regression with GEE methods for risk of IDH above and below 27°C with multiple criteria.

**Table S7:** Univariate and multivariate regression for risk of IDH with multiple criteria per four degrees Celsius decrease below the threshold (27°C).

**Figure S1.** Relationship between ambient temperature and risk of IDH compared with the subsets with the highest temperature (>31°C group) with multiple definitions.

**Figure S2**. Relationship between ambient temperature and mean ultrafiltration per dry weight.

|  |  | **Station code** | **Station name** | **Longitude** | **Latitude** | **Sessions** |
| --- | --- | --- | --- | --- | --- | --- |
| 1 |  | 46741 | Tainan | 120.204772 | 22.993239 | 45,009 |
| 2 |  | C0X100 | Beiqu Tainan City | 120.194228 | 23.010394 | 22,239 |
| 3 |  | 46742 | Yongkang | 120.236700 | 23.038386 | 8,886 |
| 4 |  | C0X160 | Rende | 120.257689 | 22.968258 | 7,581 |
| 5 |  | C0X190 | Anping | 120.152181 | 22.993161 | 3,940 |
| 6 |  | C0X110 | Nanqu Tainan City | 120.188378 | 22.961189 | 3,106 |
| 7 |  | C0O990 | Mamiao | 120.293525 | 22.991750 | 2,673 |
| 8 |  | C0V640 | Hunei | 120.244500 | 22.887139 | 2,650 |
| 9 |  | C0V630 | Qieding | 120.182608 | 22.906642 | 2,172 |
| 10 |  | C0X140 | Xigang | 120.203108 | 23.125608 | 1,783 |
| 11 |  | C0O950 | Annan | 120.144861 | 23.076694 | 1,755 |
| 12 |  | C0X150 | Anding | 120.227839 | 23.102614 | 1,740 |
| 13 |  | C0X170 | Guanmiao | 120.327797 | 22.963017 | 1,567 |
| 14 |  | C0O980 | Xinshi | 120.298197 | 23.061614 | 1,347 |
| 15 |  | C0V820 | Xiaolin | 120.259439 | 22.854997 | 866 |
| 16 |  | C0V400 | Agongdian | 120.294958 | 22.797078 | 789 |
| 17 |  | C0V750 | Luzhu | 120.327508 | 22.883208 | 647 |
| 18 |  | C0X060 | Xiaying | 120.256261 | 23.226950 | 644 |
| 19 |  | C0X130 | Guantian | 120.315422 | 23.193217 | 481 |
| 20 |  | C0X280 | Jiangjun | 120.135770 | 23.214999 | 430 |
| 21 |  | C0O900 | Shanhua | 120.297219 | 23.112883 | 95 |

**Table S1. The location of the extracted weather stations and session numbers of matched stations.**

**Table S2.** **Prevalence of IDH according to various definitions in primary and sensitivity analyses.**

| **Definition** | **Criteria** | **Prevalence** |
| --- | --- | --- |
| **Primary analysis** |  |  |
| Fall40 | (Pre-HD SBP - minimal intradialytic SBP)≥40 mmHg | 11.8% |
| Nadir90/100 | If pre-HD SBP>160, minimum intradialytic SBP<100 otherwise, minimum intradialytic SBP<90 mmHg | 10.4% |
| **Sensitivity analysis** |  |  |
| Fall20 | (Pre-HD SBP - minimal intradialytic SBP)≥20 mmHg | 34.9% |
| Fall30 | (Pre-HD SBP - minimal intradialytic SBP)≥30 mmHg | 20.6% |
| Nadir90 | Minimum intradialytic SBP<90mmHg | 9.9% |
| Nadir90 AND Fall30 | (Pre-HD SBP - minimal intradialytic SBP)≥30 mmHg and minimum intradialytic SBP<90mmHg | 5.9% |
| Nadir90 OR Fall30 | (Pre-HD SBP - minimal intradialytic SBP)≥30 mmHg or minimum intradialytic SBP<90 mmHg | 24.6% |

**Table S3. List of anti-hypertensive medications analyzed in the study**

| **Classification** | **Generic name** | **Classification** | **Generic name** |
| --- | --- | --- | --- |
| **ACEi/ARB** |  | **Alpha blocker** |  |
|  | azilsartan medoxomil |  | doxazosin |
|  | candesartan |  | silodosin |
|  | captopril |  | tamsulosin |
|  | enalapril |  | terazosin |
|  | imidapril | **CCB** |  |
|  | losartan/hydrochlorothiazide |  | amlodipine |
|  | olmesartan |  | diltiazem |
|  | ramipril |  | felodipine |
|  | sacubitril/valsartan |  | lercanidipine |
|  | telmisartan |  | nicardipine |
|  | valsartan |  | nifedipine |
| **Beta blocker** |  |  | nimodipine |
|  | atenolol |  | verapamil |
|  | bisoprolol | **Vasodilator** |  |
|  | carvedilol |  | hydralazine |
|  | esmolol |  | isosorbide dinitrate |
|  | labetalol |  | isosorbide mononitrate |
|  | metoprolol |  | minoxidil |
|  | nebivolol |  | sildenafil |
|  | propranolol | **Combination**^a^ |  |
|  |  |  | amlodipine/olmesartan |
|  |  |  | amlodipine/telmisartan |
|  |  |  | amlodipine/valsartan |
|  |  | **IV iron** |  |
|  |  |  | ferric hydroxide sucrose complex |

ACEi, angiotensin-converting enzyme inhibitor; ARB, angiotensin receptor blocker; CCB, calcium channel blocker.

For combination drugs, the drug would be classified according to the categories mentioned above. For example, the amlodipine/valsartan would be labeled in categories of CCB and ARB concomitantly.

**Table S4. Causal mediation of ambient temperature effect on intradialytic hypotension via ultrafiltration per dry weight**

Causal Mediation Analysis with Quasi-Bayesian Confidence Intervals

| Measure | Estimate | 95% CI Lower | 95% CI Upper | p-value |
| --- | --- | --- | --- | --- |
| ACME (control) | -0.0019 | -0.0021 | -0.0017 | <0.001 |
| ACME (treated) | -0.0019 | -0.0021 | -0.0017 | <0.001 |
| ADE (control) | -0.0064 | -0.0073 | -0.0054 | <0.001 |
| ADE (treated) | -0.0063 | -0.0072 | -0.0054 | <0.001 |
| Total Effect | -0.0083 | -0.0093 | -0.0073 | <0.001 |
| Prop. Mediated (control) | 0.2330 | 0.2135 | 0.2555 | <0.001 |
| Prop. Mediated (treated) | 0.2302 | 0.2110 | 0.2529 | <0.001 |
| ACME (average) | -0.0019 | -0.0021 | -0.0017 | <0.001 |
| ADE (average) | -0.0063 | -0.0072 | -0.0054 | <0.001 |
| **Prop. Mediated (average)** | **0.2316** | **0.2123** | **0.2540** | **<0.001** |

ACME, Average Causal Mediation Effect; ADE, Average Direct Effect; Prop. Mediated, Proportion Mediated

Sample Size Used: 106047

Simulations: 1000

**Table S4. Characteristics of HD sessions according to IDH definitions.**

|  |  |  | **Fall20** | | |  | **Fall30** | | |
| --- | --- | --- | --- | --- | --- | --- | --- | --- | --- |
| **Variable** | **Total number** | **All sessions** | **non-IDH (n=71,921)** | **IDH (n=38,479)** | **Standardized difference** |  | **non-IDH (n=87,671)** | **IDH (n=22,729)** | **Standardized difference** |
| Pre-HD 24h. mean temp.(°C), mean (SD) | 110,400 | 25.2(4.4) | 25.5(4.3) | 24.6(4.6) | 0.212 |  | 25.4(4.3) | 24.3(4.6) | 0.239 |
| Demographic data |  |  |  |  |  |  |  |  |  |
| Age (yr), mean (SD) | 110,400 | 62.9(13.7) | 62.8(13.9) | 63.1(13.3) | 0.020 |  | 62.9(13.8) | 63.3(13.3) | 0.030 |
| Male, n (%) | 110,400 | 55,644(50.4) | 34,026(47.3) | 21,618(56.2) | 0.178 |  | 42,379(48.3) | 13,265(58.4) | 0.202 |
| DM, n (%) | 110,400 | 48,180(43.6) | 27,929(38.8) | 20,251(52.6) | 0.280 |  | 35,042(40.0) | 13,138(57.8) | 0.363 |
| HTN, n (%) | 110,400 | 83,010(75.2) | 53,038(73.7) | 29,972(77.9) | 0.097 |  | 65,122(74.3) | 17,888(78.7) | 0.104 |
| CAD, n (%) | 110,400 | 27,222(24.7) | 17,963(25.0) | 9,259(24.1) | 0.021 |  | 21,930(25.0) | 5,292(23.3) | 0.040 |
| CCI, mean (SD) | 110,400 | 7.0(2.8) | 6.9(2.9) | 7.1(2.7) | 0.057 |  | 6.9(2.9) | 7.2(2.6) | 0.112 |
| Hemodialysis records |  |  |  |  |  |  |  |  |  |
| HD vintage (month), mean (SD) | 110,400 | 59(53) | 60(55) | 57(51) | 0.058 |  | 60(54) | 55(49) | 0.097 |
| UF / DW (%), mean (SD) | 108,635 | 3.76(1.80) | 3.42(1.70) | 4.40(1.80) | 0.556 |  | 3.53(1.72) | 4.66(1.82) | 0.636 |
| Pre-HD SBP (mmHg), mean (SD) | 110,395 | 136(22) | 131(21) | 146(22) | 0.726 |  | 133(21) | 150(22) | 0.841 |
| Blood flow (ml/min), mean (SD) | 110,084 | 275(36) | 274(35) | 278(36) | 0.099 |  | 275(35) | 277(37) | 0.070 |
| **Table S4. Continue.** | | | | | | | | | |
|  |  |  | **Fall20** | | |  | **Fall30** | | |
| **Variable** | **Total number** | **All sessions** | **non-IDH (n=71,921)** | **IDH (n=38,479)** | **Standardized difference** |  | **non-IDH (n=87,671)** | **IDH (n=22,729)** | **Standardized difference** |
| Dialysate flow (ml/min), mean (SD) | 110,379 | 564(113) | 555(108) | 581(119) | 0.223 |  | 558(109) | 589(121) | 0.269 |
| Dialysate sodium (mEq/L), mean (SD) | 109,769 | 140.0(0.6) | 140.0(0.7) | 140.0(0.6) | 0.031 |  | 140.0(0.6) | 140.0(0.7) | 0.031 |
| Dialysate calcium (mEq/L), n (%) | 110,384 |  |  |  |  |  |  |  |  |
| 1.8 |  | 1,490(1.3) | 841(1.2) | 649(1.7) | 0.044 |  | 1,146(1.3) | 344(1.5) | 0.017 |
| 2.5 |  | 58,946(53.4) | 37,588(52.3) | 21,358(55.5) | 0.065 |  | 46,497(53.0) | 12,449(54.8) | 0.035 |
| 3.0 |  | 42,286(38.3) | 27,930(38.8) | 14,356(37.3) | 0.031 |  | 33,719(38.5) | 8,567(37.7) | 0.016 |
| 3.5 |  | 7,662(6.9) | 5,553(7.7) | 2,109(5.5) | 0.090 |  | 6,297(7.2) | 1,365(6.0) | 0.047 |
| HB, mean (SD) | 110,374 | 10.6(1.0) | 10.5(1.0) | 10.7(1.0) | 0.173 |  | 10.6(1.0) | 10.7(1.0) | 0.182 |
| Antihypertensive medication | 107,867 |  |  |  |  |  |  |  |  |
| ACEi/ARB, n (%) |  | 27,204(25.2) | 18,711(26.0) | 8,493(22.1) | 0.092 |  | 22,193(25.3) | 5,011(22.0) | 0.077 |
| Beta blockers, n (%) |  | 31,692(29.4) | 21,453(29.8) | 10,239(26.6) | 0.072 |  | 25,482(29.1) | 6,210(27.3) | 0.039 |
| Alpha blockers, n (%) |  | 11,912(11.0) | 8,549(11.9) | 3,363(8.7) | 0.104 |  | 9,978(11.4) | 1,934(8.5) | 0.096 |
| **Table S4. Continue.** | | | | | | | | | |
|  |  |  | **Fall20** | | |  | **Fall30** | | |
| **Variable** | **Total number** | **All sessions** | **non-IDH (n=71,921)** | **IDH (n=38,479)** | **Standardized difference** |  | **non-IDH (n=87,671)** | **IDH (n=22,729)** | **Standardized difference** |
| Calcium channel blocker, n (%) |  | 40,708(37.7) | 29,268(40.7) | 11,440(29.7) | 0.231 |  | 34,267(39.1) | 6,441(28.3) | 0.229 |
| Vasodilator, n (%) |  | 8,712(8.1) | 6,262(8.7) | 2,450(6.4) | 0.089 |  | 7,377(8.4) | 1,335(5.9) | 0.099 |
| IV_iron, n (%) | 110,400 | 1,569(1.4) | 1,043(1.5) | 526(1.4) | 0.008 |  | 1,265(1.4) | 304(1.3) | 0.009 |

**Table S4. Continue.**

|  | **Nadir90** | | |  | **Nadir90 AND Fall30** | | |  | **Nadir90 OR Fall30** | | |
| --- | --- | --- | --- | --- | --- | --- | --- | --- | --- | --- | --- |
| **Variable** | **non-IDH (n=99,447)** | **IDH (n=10,953)** | **Standardized difference** |  | **non-IDH (n=103,917)** | **IDH (n=6,483)** | **Standardized difference** |  | **non-IDH (n=83,203)** | **IDH (n=27,197)** | **Standardized difference** |
| Pre-HD 24h. mean temp.(°C), mean (SD) | 25.2(4.4) | 24.7(4.6) | 0.116 |  | 25.2(4.4) | 24.3(4.7) | 0.210 |  | 25.4(4.3) | 24.5(4.6) | 0.203 |
| Demographic data |  |  |  |  |  |  |  |  |  |  |  |
| Age (yr), mean (SD) | 62.3(13.6) | 68.9(13.3) | 0.495 |  | 62.7(13.7) | 67.3(13.5) | 0.339 |  | 62.4(13.7) | 64.6(13.5) | 0.161 |
| Male, n (%) | 51,873(52.2) | 3,771(34.4) | 0.364 |  | 52,836(50.8) | 2,808(43.3) | 0.151 |  | 41,418(49.8) | 14,226(52.3) | 0.051 |
| DM, n (%) | 42,572(42.8) | 5,608(51.2) | 0.169 |  | 44,419(42.7) | 3,761(58.0) | 0.309 |  | 33,197(39.9) | 14,983(55.1) | 0.308 |
| HTN, n (%) | 76,047(76.5) | 6,963(63.6) | 0.284 |  | 78,380(75.4) | 4,630(71.4) | 0.091 |  | 62,793(75.5) | 20,217(74.3) | 0.026 |
| CAD, n (%) | 24,241(24.4) | 2,981(27.2) | 0.065 |  | 25,628(24.7) | 1,594(24.6) | 0.002 |  | 20,543(24.7) | 6,679(24.6) | 0.003 |
| CCI, mean (SD) | 6.9(2.8) | 8.1(2.7) | 0.426 |  | 6.9(2.8) | 7.9(2.8) | 0.333 |  | 6.9(2.9) | 7.4(2.6) | 0.188 |
| Hemodialysis records |  |  |  |  |  |  |  |  |  |  |  |
| HD vintage (month), mean (SD) | 59(53) | 62(59) | 0.065 |  | 59(53) | 55(53) | 0.075 |  | 60(54) | 58(52) | 0.028 |
| UF / DW (%), mean (SD) | 3.67(1.77) | 4.61(1.83) | 0.521 |  | 3.68(1.76) | 5.16(1.80) | 0.833 |  | 3.52(1.73) | 4.52(1.81) | 0.566 |
| Pre-HD SBP (mmHg), mean (SD) | 138(21) | 119(26) | 0.835 |  | 136(23) | 134(20) | 0.102 |  | 135(20) | 146(29) | 0.285 |
| Blood flow (ml/min), mean (SD) | 277(35) | 260(33) | 0.510 |  | 276(35) | 263(35) | 0.363 |  | 276(35) | 274(37) | 0.067 |
| Dialysate flow (ml/min), mean (SD) | 566(114) | 543(97) | 0.220 |  | 564(113) | 558(107) | 0.054 |  | 560(111) | 578(118) | 0.157 |
| Dialysate avg. temp.(°C), mean (SD) | 36.1(0.4) | 35.9(0.4) | 0.460 |  | 36.1(0.4) | 35.9(0.4) | 0.472 |  | 36.1(0.4) | 36.0(0.4) | 0.256 |
| **Table S4. Continue.** |  |  |  |  |  |  |  |  |  |  |  |
|  | **Nadir90** | | |  | **Nadir90 AND Fall30** | | |  | **Nadir90 OR Fall30** | | |
| **Variable** | **non-IDH (n=99,447)** | **IDH (n=10,953)** | **Standardized difference** |  | **non-IDH (n=103,917)** | **IDH (n=6,483)** | **Standardized difference** |  | **non-IDH (n=83,203)** | **IDH (n=27,197)** | **Standardized difference** |
| Dialysate sodium (mEq/L), mean (SD) |  |  |  |  |  |  |  |  |  |  |  |
| Dialysate calcium (mEq/L), n (%) | 140.0(0.7) | 140.0(0.5) | 0.033 |  | 140.0(0.7) | 140.0(0.5) | 0.052 |  | 140.0(0.7) | 140.0(0.6) | 0.031 |
| 1.8 | 1430(1.4) | 60(0.5) | 0.090 |  | 1457(1.4) | 33(0.5) | 0.092 |  | 1119(1.3) | 371(1.4) | 0.002 |
| 2.5 | 52,938(53.2) | 6,008(54.9) | 0.033 |  | 55,348(53.3) | 3,598(55.5) | 0.045 |  | 44,087(53.0) | 14,859(54.6) | 0.033 |
| 3.0 | 37,976(38.2) | 4,310(39.3) | 0.024 |  | 39,813(38.3) | 2,473(38.1) | 0.003 |  | 31,884(38.3) | 10,402(38.2) | 0.002 |
| 3.5 | 7088(7.1) | 574(5.2) | 0.078 |  | 7,283(7.0) | 379(5.8) | 0.047 |  | 6,102(7.3) | 1,560(5.7) | 0.065 |
| HB, mean (SD) | 10.6(1.0) | 10.7(1.0) | 0.146 |  | 10.6(1.0) | 10.7(1.0) | 0.138 |  | 10.6(1.0) | 10.7(1.0) | 0.190 |
| Antihypertensive medication |  |  |  |  |  |  |  |  |  |  |  |
| ACEi/ARB, n (%) | 26,343(26.5) | 861(7.9) | 0.510 |  | 26,504(25.5) | 700(10.8) | 0.389 |  | 22,031(26.5) | 5,173(19.0) | 0.179 |
| Beta blockers, n (%) | 29,891(30.1) | 1,801(16.4) | 0.327 |  | 30,327(29.2) | 1,365(21.1) | 0.188 |  | 25,045(30.1) | 6,647(24.4) | 0.127 |
| Alpha blockers, n (%) | 11,575(11.6) | 337(3.1) | 0.332 |  | 11,633(11.2) | 279(4.3) | 0.260 |  | 9,920(11.9) | 1,992(7.3) | 0.156 |
| Calcium channel blocker, n (%) | 39,725(39.9) | 983(9.0) | 0.772 |  | 39,879(38.4) | 829(12.8) | 0.613 |  | 34,112(41.0) | 6,596(24.3) | 0.363 |
| Vasodilator, n (%) | 8,306(8.4) | 406(3.7) | 0.196 |  | 8,450(8.1) | 262(4.0) | 0.172 |  | 7,232(8.7) | 1,480(5.4) | 0.127 |
| IV_iron, n (%) | 1,465(1.5) | 104(0.9) | 0.055 |  | 1,497(1.4) | 72(1.1) | 0.027 |  | 1,233(1.5) | 336(1.2) | 0.026 |

* Data presented as mean (SD) in the continuous variables, and numbers (n) with the percentage (%) in categorical variables. Temp., temperature; DM, diabetes mellitus; HTN, hypertension; CAD, coronary artery disease; CCI, Charlson Comorbidity Index; HD, hemodialysis; UF/DW, ultrafiltration weight to dry weight ratio; SBP, systolic blood pressure; avg, average; Hb, hemoglobin; ACEi, angiotensin-converting enzyme inhibitor; ARB, angiotensin receptor blocker; IV_iron, intravenous iron infusion. A standardized difference of < 0.1 was considered a negligible difference between groups.

**Table S5.** **Logistic regression with GEE methods for risk of IDH above and below 27°C with multiple criteria**

|  |  | **Overall** | **Stratified by temperature** | | ***P*_interaction_^b^** |
| --- | --- | --- | --- | --- | --- |
|  |  |  | **≦27℃** | **>27℃** |  |
| **Definition** |  | **(n=105,895)^a^** | **(n=59,751)^a^** | **(n=46,144)^a^** |  |
| Fall20 | Adjusted OR^c^ | 1.166 | 1.214 | 0.943 |  |
|  | 95% CI | 1.122 to 1.211 | 1.169 to 1.260 | 0.837 to 1.063 |  |
|  | *P* value | <0.001 | <0.001 | 0.34 | <0.001 |
|  |  |  |  |  |  |
| Fall30 | Adjusted OR^c^ | 1.190 | 1.235 | 0.893 |  |
|  | 95% CI | 1.145 to 1.237 | 1.188 to 1.284 | 0.778 to 1.025 |  |
|  | *P* value | <0.001 | <0.001 | 0.11 | <0.001 |
|  |  |  |  |  |  |
| Nadir90 | Adjusted OR^c^ | 1.178 | 1.209 | 0.966 |  |
|  | 95% CI | 1.121 to 1.238 | 1.149 to 1.272 | 0.799 to 1.167 |  |
|  | *P* value | <0.001 | <0.001 | 0.72 | 0.010 |
|  |  |  |  |  |  |
| Nadir90 AND Fall30 | Adjusted OR^c^ | 1.193 | 1.252 | 0.974 |  |
|  | 95% CI | 1.130 to 1.260 | 1.183 to 1.325 | 0.775 to 1.224 |  |
|  | *P* value | <0.001 | <0.001 | 0.82 | 0.026 |
|  |  |  |  |  |  |
| Nadir90 OR Fall30 | Adjusted OR^c^ | 1.167 | 1.193 | 0.931 |  |
|  | 95% CI | 1.128 to 1.207 | 1.152 to 1.234 | 0.821 to 1.055 |  |
|  | *P* value | <0.001 | <0.001 | 0.26 | <0.001 |

^a^ The number was reduced because of missing data.

^b^ Interaction for ambient temperature and temperature above/below the threshold 27°C.

c Adjusted for age, sex, diabetes, coronary artery disease, Charlson index modified, HD vintage, ultrafiltration to dry weight ratio, pre-HD SBP, blood flow, dialysate flow, dialysate average temperature, dialysate sodium, dialysate calcium, hemoglobin, antihypertensive medications and iron infusion.

**Table S6.** **Univariate and multivariate regression for risk of IDH with multiple criteria per four degrees Celsius decrease below the threshold (27°C)**

| **Definition** |  | **Univariate** | **Model 1^a^** | **Model 2^b^** | **Model 3^c^** |
| --- | --- | --- | --- | --- | --- |
| Fall20 | OR | 1.204 | 1.212 | 1.196 | 1.214 |
|  | 95% CI | 1.165 to 1.244 | 1.172 to 1.254 | 1.154 to 1.239 | 1.169 to 1.260 |
|  | *P* value | <0.001 | <0.001 | <0.001 | <0.001 |
|  |  |  |  |  |  |
| Fall30 | OR | 1.221 | 1.232 | 1.220 | 1.235 |
|  | 95% CI | 1.178 to 1.266 | 1.188 to 1.278 | 1.176 to 1.267 | 1.188 to 1.284 |
|  | *P* value | <0.001 | <0.001 | <0.001 | <0.001 |
|  |  |  |  |  |  |
| Nadir90 | OR | 1.114 | 1.112 | 1.187 | 1.209 |
|  | 95% CI | 1.069 to 1.160 | 1.064 to 1.162 | 1.131 to 1.245 | 1.149 to 1.272 |
|  | *P* value | <0.001 | <0.001 | <0.001 | <0.001 |
|  |  |  |  |  |  |
| Nadir90 AND Fall30 | OR | 1.201 | 1.200 | 1.236 | 1.252 |
|  | 95% CI | 1.139 to 1.267 | 1.137 to 1.267 | 1.171 to 1.306 | 1.183 to 1.325 |
|  | *P* value | <0.001 | <0.001 | <0.001 | <0.001 |
|  |  |  |  |  |  |
| Nadir90 OR Fall30 | OR | 1.189 | 1.194 | 1.175 | 1.193 |
|  | 95% CI | 1.149 to 1.230 | 1.154 to 1.236 | 1.137 to 1.215 | 1.152 to 1.234 |
|  | *P* value | <0.001 | <0.001 | <0.001 | <0.001 |

^a^ Model 1 adjusted for age, sex, diabetes, hypertension, coronary artery disease, and Charlson index modified.

^b^ Model 2 adjusted for covariates in model 1 and additionally adjusted for HD vintage, ultrafiltration weight to dry weight ratio (UF/DW), pre-HD SBP, blood flow, dialysate flow, dialysate average temperature and dialysate calcium.

^c^ Model 3 further adjusted for antihypertensive medications and iron infusion but omitted covariate of hypertension to avoid collinearity.


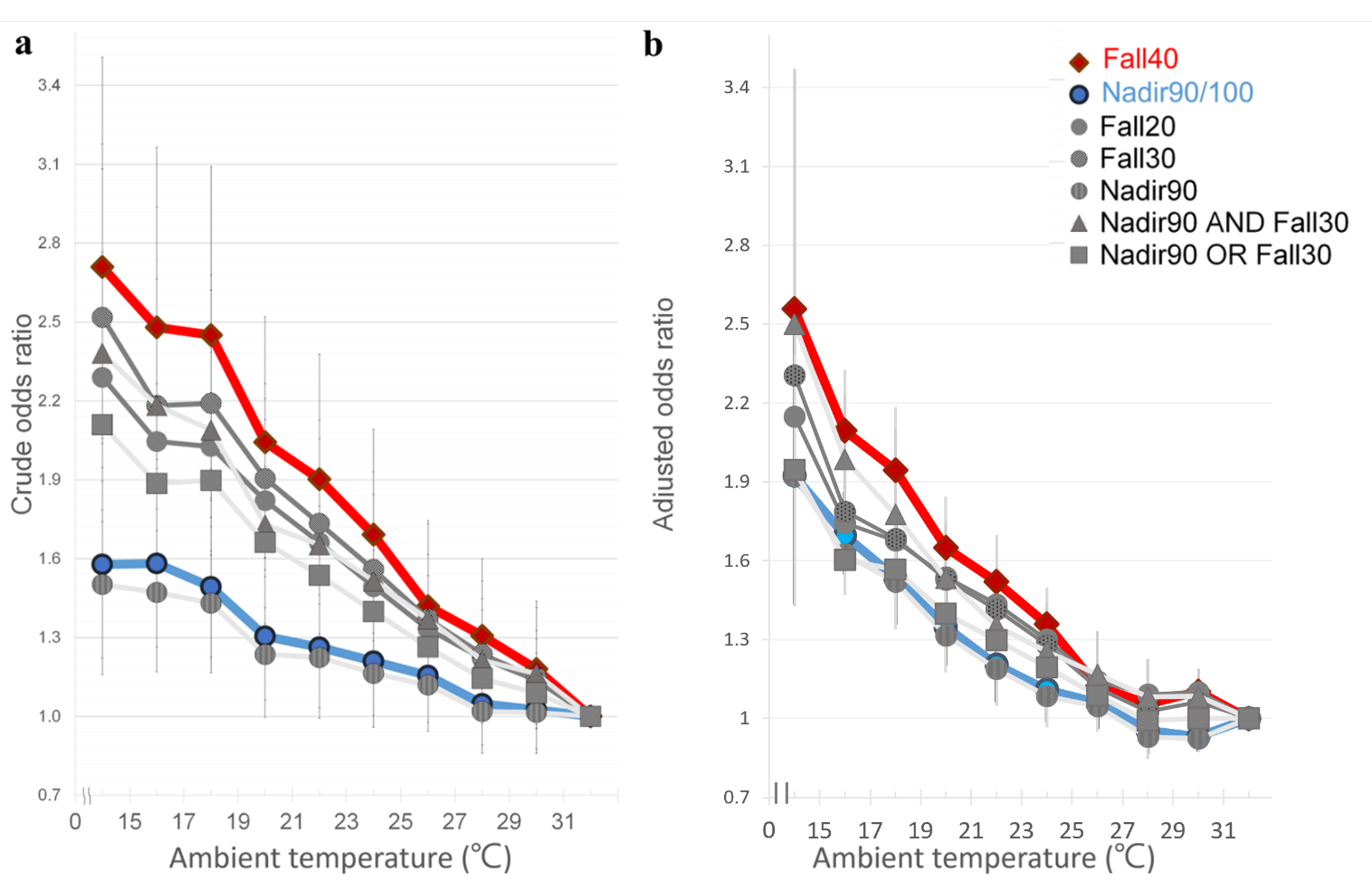


**Figure S1. Relationship between ambient temperature and risk of IDH compared with the subsets with the highest temperature (>31°C group) (a) crude odds ratio (b) adjusted odds ratio with adjustment of age, sex, diabetes, coronary artery disease, ultrafiltration to dry weight ratio, pre-HD SBP, blood flow, dialysate flow, dialysate mean temperature and dialysate calcium, and antihypertensive medications.**


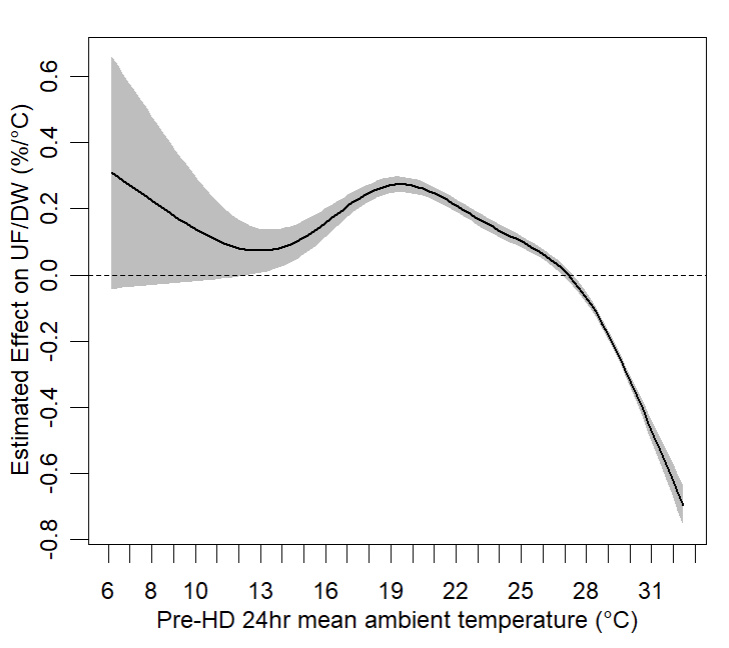


**Figure S2. Generalized Additive Model Analysis of Ambient Temperature's Effect on Ultrafiltration per Dry Weight (UF/DW) with Covariate Adjustment.**
